# Evidence for the Mitochondrial Lactate Oxidation Complex in Rat Neurons: Demonstration of an Essential Component of Brain Lactate Shuttles

**DOI:** 10.1371/journal.pone.0002915

**Published:** 2008-08-13

**Authors:** Takeshi Hashimoto, Rajaa Hussien, Hyung-Sook Cho, Daniela Kaufer, George A. Brooks

**Affiliations:** Department of Integrative Biology, University of California, Berkeley, California, United States of America; National Institutes of Health, United States of America

## Abstract

To evaluate the presence of components of a putative Intracellular Lactate Shuttle (ILS) in neurons, we attempted to determine if monocarboxylate (e.g. lactate) transporter isoforms (MCT1 and -2) and lactate dehydrogenase (LDH) are coexpressed in neuronal mitochondria of rat brains. Immunohistochemical analyses of rat brain cross-sections showed MCT1, MCT2, and LDH to colocalize with the mitochondrial inner membrane marker cytochrome oxidase (COX) in cortical, hippocampal, and thalamic neurons. Immunoblotting after immunoprecipitation (IP) of mitochondria from brain homogenates supported the histochemical observations by demonstrating that COX coprecipitated MCT1, MCT2, and LDH. Additionally, using primary cultures from rat cortex and hippocampus as well as immunohistochemistry and immunocoprecipitation techniques, we demonstrated that MCT2 and LDH are coexpressed in mitochondria of cultured neurons. These findings can be interpreted to mean that, as in skeletal muscle, neurons contain a mitochondrial lactate oxidation complex (mLOC) that has the potential to facilitate both intracellular and cell-cell lactate shuttles in brain.

## Introduction

Lactate is produced continuously under fully aerobic conditions in mammalian skeletal muscle, especially during exercise when rates of glycogenolysis and glycolysis are elevated [1], [2]. In addition to lactate production, working skeletal muscles are also capable of lactate removal, mainly via oxidation [3], [4]. Consequently, the lactate shuttle concept has been postulated to contain both cell-cell [5] and intracellular components [1].

The concept of lactate shuttles as means to distribute potential energy and provide a redox signaling mechanism within and among cells [6] has been extended to the field of neuroscience [7], [8], [9]. The astrocyte-neuron lactate shuttle hypothesis (ANLS) posits that lactate is an essential element of neuron-glia metabolic interactions [8], [10]. Due to the ready accessibility to the vasculature and the feasibility of muscle biopsy techniques, the presence of a Cell-Cell Lactate Shuttle (CCLS) [6] has been extensively supported in the periphery [1], [11]. However, limited access to the cerebral circulation and limited capacity for tissue sampling has made evaluation of lactate shuttling within and among brain cells difficult. Still, with current technologies it is possible to evaluate components of Cell-Cell and Intracellular Lactate Shuttles in brain. In the present study, we attempted to determine if a Mitochondrial Lactate Oxidation Complex (mLOC) exists in rat brain as it does in rodent [12], [13] and human [14] skeletal muscle. By using confocal laser scanning microscopy (CLSM) and immunoblotting after immunoprecipitation from cell lysates, we demonstrated that MCT1, MCT2, and LDH are located in neuronal mitochondria, and additionally that MCTs and LDH are associated with cytochrome oxidase (COX) in rat brain mitochondria.

## Methods

### Materials

Aprotinin, DTT, EDTA, EGTA, HEPES, Leupeptin, Mops, Nonidet P-40 (NP-40), Pepstatin A, PMSF, Sucrose, Tris, cytosine-β-D-arabinofuranoside (Ara-C), and ε-aminocaproic acid were purchased from Sigma-Aldrich (St. Louis, MO). NaCl and NaN_3_ were purchased from Fisher (Fairlawn, NJ). Na_4_P_2_O_7_⋅10 H_2_O was purchased from Matheson Coleman & Bell (Norwood, OH). Tissue culture reagents were purchased from Invitrogen (Grand Island, NY).

### Animal care and tissue sampling

The University of California, Berkeley ACUC approved all protocols. Female Wistar rats (200–250 g) were fed and housed under standard conditions. Animals were anesthetized via pentobarbital sodium injection (50 mg/kg ip). For biochemistry, whole brains were dissected, immediately frozen in liquid nitrogen, ground into powder, and stored at −80°C until analysis. For immunohistochemistry, rats were anesthetized and intracardially perfused with 500 ml of normal saline at room temperature, followed by 500 ml of ice-cold, freshly made 4% paraformaldehyde in phosphate buffer (PB, 0.1 M, pH 7.4). For immunolabeling, brains were removed, post-fixed for 4 hours in 4% paraformaldehyde at 4°C and then cryoprotected in 20% sucrose at 4°C for at least 1 day; sections (40 µm) were cut on a microtone and collected in cold PB.

### Rat Mesencephalic Neuron-Glia Cultures

Primary hippocampus and cortex neuron-glia cultures were prepared from the brains of embryonic day-18 Wistar rats as previously described [15], [16]. Brains were removed aseptically and the hippocampus and cortex were dissected. After removing blood vessels and meninges, hippocampal and cortical tissues were dissociated by mild mechanical trituration in ice-cold calcium- and magnesium-free Hank's balanced salt solution (HBSS) with 10 mM HEPES and 20 mM glucose, pH 7.4. Cells were freed by digestion in a papain solution (100 U/10 ml) for 20 min and the reaction was stopped by adding 10% horse serum. After pelleting by centrifugation, cells from the two brain areas were resuspended and plated (1.5 10^5^/1 ml medium/well) to 24-well cell culture plates or (7.0 10^5^/10 ml medium/dish) to 10 cm dishes precoated with poly-L-lysine (500 µg/ml). The minimum essential culture medium was supplemented with 5.4% (not heat-inactivated) fetal bovine serum (FBS), 0.1% serum extender, and 23 mM glucose. Separate cultures derived from hippocampus and cortex were maintained at 37°C in a humidified atmosphere of 5% CO_2_, 95% air. Seven days later, 1 µl/ml cytosine-β-D-arabinofuranoside (Ara-C) with fresh Neurobasal media (with 2% B27 supplement and 25% Glutamax) was added to the cultures. Cell harvesting for immunohistochemistry and other procedures was conducted days 10–14.

### Immunohistochemical analyses

For immunolabeling, rat brain cross-sections were pretreated in 50% ethanol and incubated in 10% normal donkey serum for 40 minutes. For immunolabeling, primary cells were fixed with ice-cold acetone for 5 min and then permeabilized with 0.2% Triton X-100 in PBS for 5 min. Thereafter, sections or primary cells were incubated overnight at 4°C with rabbit anti-MCT1 (Brooks, custom antibody), rabbit anti-MCT2 (Chemicon International, Temecula, CA), mouse anti-cytochrome oxidase subunit IV (COX: Abcam, Cambridge, MA), rabbit anti-voltage-dependent anion channel (VDAC: Abcam), mouse anti-LDH H or M (Sigma-Aldrich, St. Louis, MO), goat or rabbit anti-LDH (Abcam), chicken anti-microtubule-associated protein (MAP2: Abcam), mouse anti-neuron specific beta III Tublin (Tuj1: Covance), or chicken anti-glial fibrillary acidic protein (GFAP: Abcam). In vivo, the five isoforms of LDH exist as combinations of two subtypes: H (predominant in heart) and M (predominant in liver and skeletal muscle), encoded by two different genes. The M (also known as A or 5) subunit is encoded on chromosome 11p15.4, whereas the H (aka B or 1) subunit is encoded on chromosome 12p12.2-p12.1. For clarity we shall use “H” and “M” terminology as it has some meaning for tissue LDH isoform expression in nature. Anti-rabbit secondary antibodies conjugated to Alexa Fluor 488 were used for MCT1, MCT2, and VDAC detection. Anti-goat Alexa Fluor 546 secondary antibodies (Molecular Probes) were used for LDH detection. For COX, anti-mouse Cy3 (Chemicon International) was used. Anti-chicken Cy5 (Abcam) was used for MAP2 detection. Anti-chicken Alexa Fluor 488 (Molecular Probes) was used for GFAP detection. Secondary antibodies were incubated for 1 hr at room temperature. Confocal laser scanning microscopy (CLSM: Zeiss 510 META) was used for immunofluorescent detection of cellular localizations of the mitochondrial reticulum (COX, VDAC), MCT1, MCT2 and LDH as previously described [13]. Antibody staining was detected at an emission of 500–530 nm, 550–600 nm, and 640–720 nm, after excitation at 488 nm, 543 nm, and 630 nm, respectively. An oil immersion objective (Zeiss 40×/1.3 N.A. or 63×/1.4 N.A.) was used to visualize stained sections. Images represent optical slices of ∼1.1 µm, and laser power and detection gains were set such that signals from single-stained controls would not appear in adjacent channels.

### Preparation of subcellular fractions

After harvesting with PBS, primary cells were collected by centrifuging at 700 g for 10 min at 4°C. Primary cells or powdered brain tissues were gently homogenized in buffer A (250 mM Sucrose, 5 mM NaN_3_, 2 mM EGTA, 100 µM PMSF, 1 µM Pepstatin A, 10 µM Leupeptin, 20 mM HEPES-Na, pH 7.4) using a loose-fitting Dunce (Teflon-glass) homogenizer, and then centrifuged at 600 g for 10 min at 4°C to remove nuclei, debris, and unbroken cells. The supernatant was centrifuged at 10,000 g for 30 min at 4°C to precipitate mitochondria. The pellet from this high-speed spin was washed in buffer B (1 mM EDTA and 10 mM Tris, pH 7.4) and then resuspended in 200 µL of buffer C (10 mM Tris, 1 mM EDTA, 150 mM NaCl, and 10 µg/ml aprotinin, pH 7.4) with 4% SDS and centrifuged at room temperature at 1,100 g for 20 min. This supernatant was used for immunoblots of mitochondrial (MI) fraction. The rest of the mitochondrial pellet was washed three times in buffer B and resuspended in 500 µL of buffer C with 0.3–1% Tween 20; this mitochondrial fraction was used for immunoprecipitation (IP).

### Immunoprecipitation (IP)

Immunoprecipitation was conducted as previously described [13]. Agarose-conjugated protein G (Santa Cruz Biotechnology) was added to the mitochondrial fractions and incubated at 4°C for 30 min. Samples were centrifuged at 2300 g and 4°C for 1 min and aliquots of the supernatant were reacted with primary antibodies to COX and rat normal IgG (Santa Cruz) at 4°C for overnight. Then, agarose-conjugated protein G was added and incubated at 4°C for 1 hr. The pellet was collected by centrifugation at 2300 g at 4°C for 1 min. The supernatant (lysate) was used for SDS-PAGE. The pellet was washed 3 times by PBS buffer (pH 7.4) and 1 time by buffer C. The final pellet from immunoprecipitation (IP) was used for SDS-PAGE.

### Immunoblots (IB)

The presence of MCT1, MCT2, and LDH in neuronal mitochondrial fractions was determined by standard Western blotting techniques [13]. The antibodies to MCT1, MCT2, and LDH were the same as described in the CLSM procedure. In addition, identity of the mitochondrial fraction was confirmed by probing for COX. Evaluation of contamination of the mitochondrial fraction by sarcolemmal remnants was done by probing with rabbit anti-β_1_-Na^+^-K^+^-ATPase (Upstate Serologicals, Charlottesville, VA), rabbit anti-GLUT1 (Santa Cruz), and F-actin (Abcam). MagicMark XP Western Standard (Invitrogen, Grand Island, NY) was used for molecular weight standards.

## Results

### Mitochondrial MCT1, MCT2, and LDH detected by immunohistochemistry in rat brain tissues

**Figure 1:**
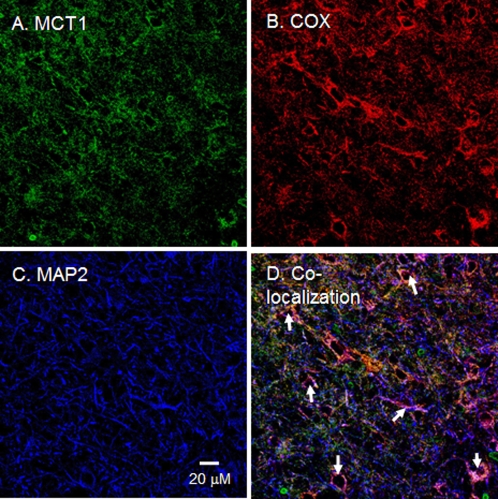
Immunohistochemical images of rat brain cross-sections demonstrating mitochondrial MCT1 in thalamic neurons. When signals from probes for the lactate/pyruvate transporter MCT1 (A, green) and mitochondrial cytochrome oxidase, COX (B, red) were merged with those of the neuronal marker MAP2 (C, blue), superposition of the signals (D, yellow/white) showed colocalization of MCT1 and components of the mitochondrial reticulum in neurons (white arrows). Scale bar = 20 µm.

**Figure 2 pone-0002915-g002:**
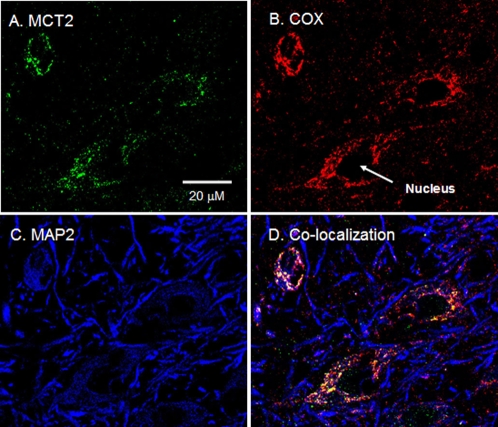
Immunohistochemical images of rat brain cross-sections demonstrating mitochondrial MCT2 in thalamic neurons. When signals from probes for MCT2 (A, green), and mitochondrial COX (B, red) were merged with those of the neuronal marker MAP2 (C, blue), superposition of the signals (D, yellow/white) showed colocalization of MCT2 and components of the mitochondrial reticulum in neurons (white arrows). Scale bar = 20 µm.

**Figure 3 pone-0002915-g003:**
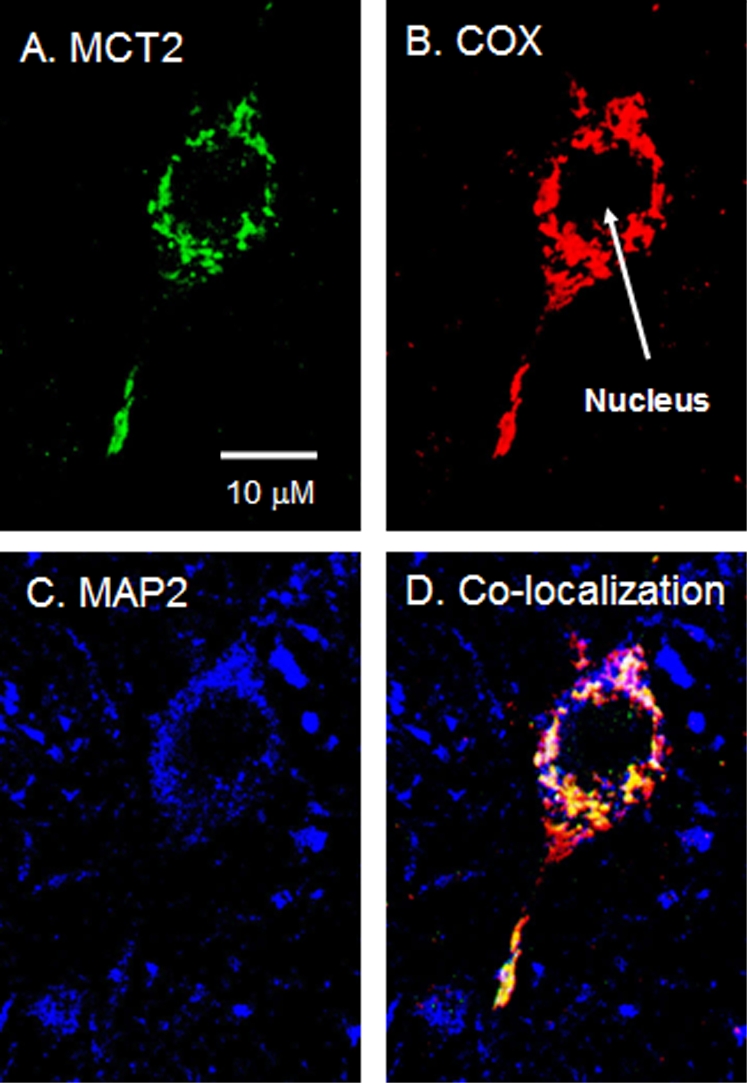
Immunohistochemical images of rat brain cross-sections demonstrating mitochondrial MCT2 in hippocampal neurons. Superposition of signals from probes for MCT2 (A, green), COX (B, red) and MAP2 (C, blue) showed clear colocalization (D, yellow/white) in neurons. Scale bar = 10 µm.

MCT1 and MCT2 were detected by CLSM mainly in the thalamus and hippocampus of rat brain tissues (Figs. 1–3). Fig. 1 shows the presence of MCT1, the mitochondrial marker COX, and the neuronal marker MAP2 in rat thalamus. Comparing staining for MCT1 (Fig. 1A) or COX (Fig. 1B) with MAP2 (Fig. 1C), it appears that MCT1 and mitochondria are abundant in neurons. Furthermore, the superposition of the probes for MCT1, COX, and MAP2 clearly shows colocalization of MCT1 with the mitochondrial reticulum of neurons (Fig. 1D). In addition to the result with MCT1, we detected MCT2 in neurons of the rat thalamus (Fig 2). There, superposition of probes for MCT2, COX, and MAP2 also clearly shows mitochondrial MCT2 in neurons (Fig. 2D). Fig. 3 represents a neuron in CA2 of the hippocampus; at this larger magnification, the presence of MCT2 in the mitochondrial reticulum of a neuron is evident (Fig. 3D).

To assess specificity of brain area LDH isoform expression, we probed for LDH-M (as typically found in skeletal muscle) and LDH-H (as typically found in heart) in the hippocampus, thalamus and entorhinal and somatosensory rat cortex.

**Figure 4 pone-0002915-g004:**
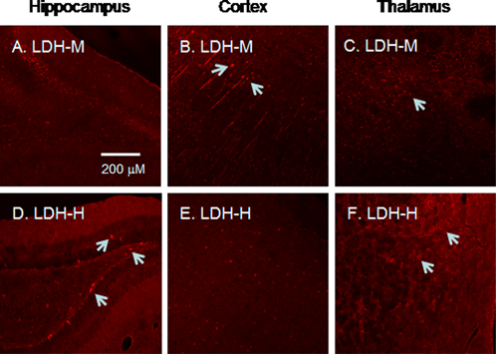
Immunohistochemical images of rat brain cross-sections showing area-specific expression of LDH-M (A–C) and LDH-H (D–F). In hippocampus (A and D), signals for LDH-H predominate (D, arrows) whereas a signal for LDH-M is not evident (A). On the other hand, in cortex (B and E), signals for LDH-M predominate (B, arrows), but a signal for LDH-H is not evident (E). In thalamus (C and F), signals for LDH-H predominate (F, arrows), but a signal for LDH-M is also apparent (C, arrow). Scale bar = 200 µm.

**Figure 5 pone-0002915-g005:**
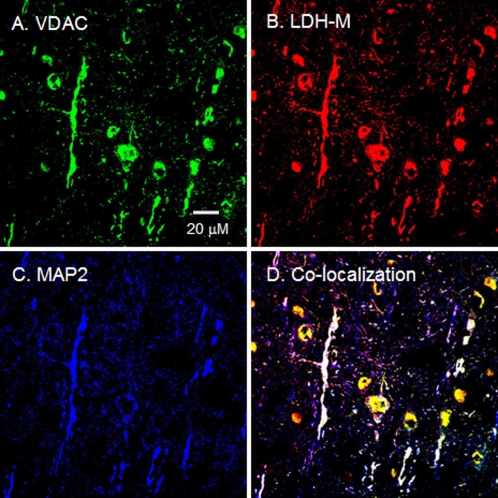
Immunohistochemical images of rat brain demonstrating mitochondrial LDH-M in cortical neurons. When signals from probes for LDH-M (B, red) and the mitochondrial protein VDAC (A, green) were merged with those for the neuronal marker MAP2 (C, blue), superposition of the signals (D, yellow/white) showed colocalization of LDH-M and components of the mitochondrial reticulum. Scale bar = 20 µm.

**Figure 6:**
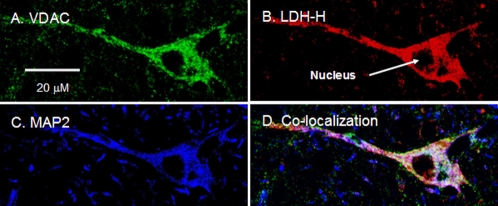
Immunohistochemical images of rat brain cross-sections demonstrating mitochondrial LDH-H in hippocampal neurons. Superposition of signals from probes for VDAC (A, green), LDH-H (B, red) and MAP2 (C, blue) showed extensive colocalization throughout the neuron (D, yellow/white). Scale bar = 20 µm.

LDH-M protein expression was clearly detected in rat cortex (Fig. 4B), and merged images indicated the pattern of LDH-M localization to match that of the mitochondrial marker VDAC (Fig. 5). On the other hand, LDH-H protein expression was clear in neurons of the rat hippocampus (Fig. 4D). In the micrograph with larger magnification, superposition of signals for LDH-H and VDAC clearly showed LDH to colocalize with the mitochondrial reticulum (white in Fig. 6D). In rat thalamus, signals for LDH-H predominate (Fig. 4F), but a signal for LDH-M is also apparent (Fig. 4C).

### MCT1 and MCT2 are associated with COX in rat brain tissue

**Figure 7:**
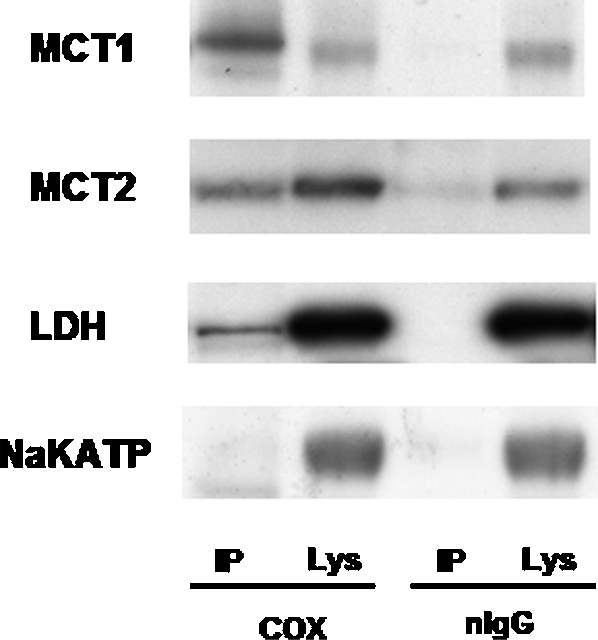
Antibodies to COX and normal IgG (nIgG) were used to develop immunoprecipitates (IP) from Tween 20 (1%)-solubilized mitochondrial fractions of brain tissue. IP proteins were subsequently probed with antibodies to MCT1, MCT2, LDH, and β_1_-Na^+^-K^+^-ATPase. MCT1 and MCT2 were coprecipitated with COX, and LDH was slightly, but significantly coprecipitated with COX as compared to nIgG (no protein was coprecipitated with nIgG). The plasma membrane marker β_1_-Na^+^-K^+^-ATPase was not coprecipitated with either COX or nIgG. Sup, supernatant after immunoprecipitation in lysates of mitochondrial fractions.

In order to determine the subcellular localization and the physical association between the various components of the ILS, we performed immunocoprecipitation experiments on solubilized mitochondrial fractions. Protein complexes in mitochondrial fractions were pulled down with antibodies to either the mitochondrial protein COX or normal IgG (nIgG) as a control, and then probed for MCT1, MCT2, LDH and the plasma membrane marker protein Na^+^/K^+^-ATPase (Fig. 7). COX coprecipitated MCT1, MCT2, and to a lesser extent, LDH from the mitochondrial fraction of rat brain tissue homogenates. However, COX did not coprecipitate Na^+^/K^+^-ATPase. The specificity of the complex composition was further demonstrated by the absence of coprecipitation of these proteins with nIgG. When isolating brain mitochondrial fractions, we avoided the use of procedures known to cause loss of proteins associated with COX and LDH such as proteases, detergents and Percoll gradients [12], [17], [18]. Therefore, the mitochondrial fractions we obtained from brain contained remnants of cytoskeleton and plasma membrane constituents (such as Na^+^-K^+^-ATPase) that are highly expressed in brain cells [18]. Separation of mitochondrial reticulum remnants from non-mitochondrial cell constituents is known to be difficult [19], but because the plasma membrane marker Na^+^-K^+^-ATPase was not detected in immunoprecipitates, contamination was unlikely to be a confounding factor in the interpretation of results.

### Mitochondrial MCT2 and LDH detected by immunocytochemistry in rat brain primary cell cultures

**Figure 8:**
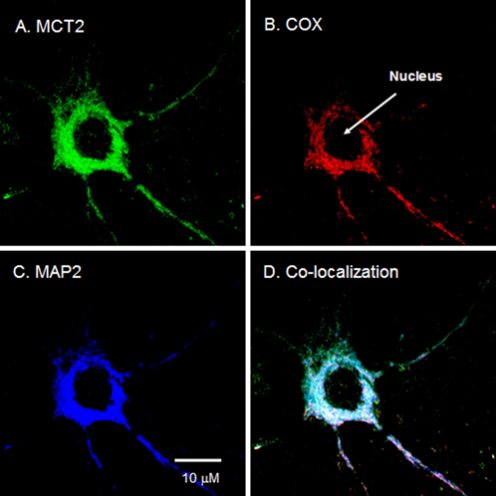
Immunohistochemical images demonstrating mitochondrial MCT2 in a cultured neuron from rat hippocampus. Superposition of signals from probes for MCT2 (A, green), COX (B, red), and MAP2 (C, blue) showed extensive colocalization (D, white) in a cultured neuron. Scale bar = 10 µm.

Existence of an ANLS *in vivo* is controversial in the current literature [20], [21]. Part of the difficulty in evaluating presence of an ANLS arises because of regional neuronal heterogeneity as well as neuron-astroglia complexity, giving rise to observations of different patterns of MCT expression *in vivo* and *in vitro* [22], [23]. In an attempt to resolve interpretation of previously observed differences, we assessed the expression patterns of mitochondrial MCT and LDH isoforms in mixed neuron/glia primary cell cultures derived from rat hippocampus as well as from cortex. Cultures were enriched for neuronal development by use of the astrocyte inhibitor cytosine-β-D-arabinofuranoside (Ara-C), with the result being a composite population of >90% neurons and <10% astrocytes (data not shown). When data obtained on tissue sampled *in vivo* and cultured cells were compared, differences were obtained with regard to the expression of MCT1. Figure 8 shows the presence of mitochondrial MCT2 in a neuronal cell cultured from rat hippocampus. Similarly, neuronal MCT2 was detected in primary cells of cortex (micrographs are not shown). However, contrary to what was seen in cross-sections of adult rat brain (Fig. 1), MCT1 was not evident in primary culture cells.

**Figure 9:**
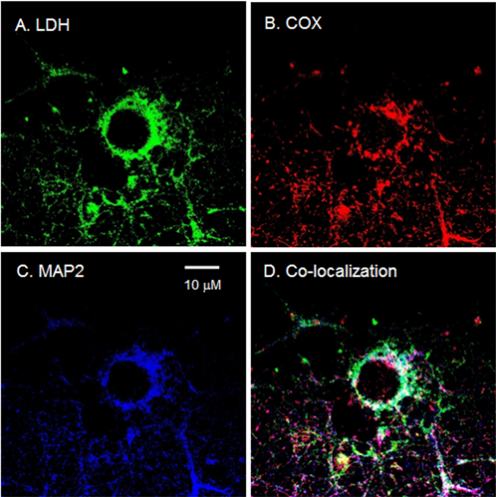
Immunohistochemical images demonstrating mitochondrial LDH in neurons cultured from rat hippocampus and cortex. Superposition of signals from probes for LDH (A, green), COX (B, red) and MAP2 (C, blue) showed clear colocalization (D, yellow/white) in neuron cells. Scale bar = 10 µm.

In addition to heterogeneity in the expression of mMCT1 and -2, we also saw differences in the distribution of mLDH when data obtained *in vivo* and *in vitro* were compared. Figure 9 shows the presence of mitochondrial LDH in a cultured neuron labeled with an antibody that recognizes all LDH isoforms. This result was typical of cells obtained from rat hippocampus and cortex. When isoform-specific antibodies were used, an antibody to LDH-M stained neurons of the cortex, but the same antibody only faintly stained neurons in the hippocampus. Those results suggest the predominance of LDH-M in rat cortical neuron mitochondria, and LDH-H in hippocampal neuron mitochondria. These latter results and our interpretation of them correspond to localizations seen in rat brain cross-sections (e.g., Fig. 4).

### Mitochondrial outer membrane association with plasma membrane Na^+^-K^+^-ATPase

**Figure 10:**
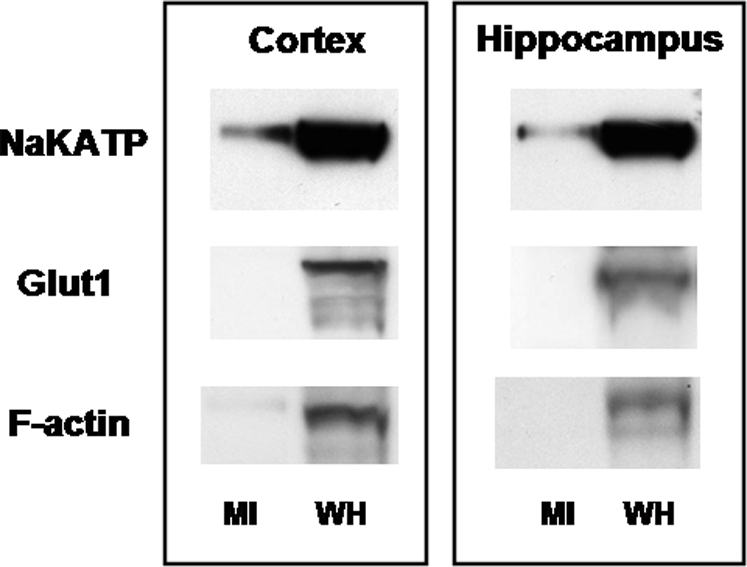
Immunoblots of mitochondrial fractions (MI) from primary neuronal cultures developed from rat cortex and hippocampus. Whole homogenates (WH) of the same cells were loaded as positive controls. Mitochondria were immunoblotted using plasma membrane markers for β_1_-Na^+^-K^+^-ATPase and GLUT1 as well as the cytosol marker F-actin. Mitochondrial fractions (MI) showed the presence of β_1_-Na^+^-K^+^-ATPase. However, while prominent in whole cell homogenates (WH), the other plasma membrane marker Glut1 and the cytosol marker F-actin were not apparent.

Results of CLSM studies on adult brain sections clearly showed LDH and MCT to colocalize with COX in mitochondria (Figs. 1–6). However, to obtain information from an independent technique, we probed primary enriched neuronal cultures. That procedure allowed for improved purification of mitochondrial fractions. Figure 10 shows immunoblots of mitochondrial fractions of cultured neuronal cells. Recalling that COX coprecipitated MCT1, MCT2 and LDH, but not Na^+^-K^+^-ATPase (Fig. 7), results in Fig. 10 are different in showing that isolated mitochondria obtained from primary neuronal cell cultures contained Na^+^-K^+^-ATPase. Consistent with recent results obtained on an astrocytoma cell line [24], the non-mitochondrial fraction markers GLUT1 and F-actin were not detected in mitochondrial fractions from either cortical and hippocampal primary culture cells (Fig. 10). Hence, rather than indicating contamination, it may be that the Na^+^-K^+^-ATPase is associated with the outer mitochondrial membrane [25]. In this way, the ATP system necessary for maintaining plasma membrane cation gradients could be associated with the system for maintaining cellular ATP homeostasis.

### Interactions of COX and LDH detected by immunocoprecipitation in rat brain primary cell cultures

**Figure 11:**
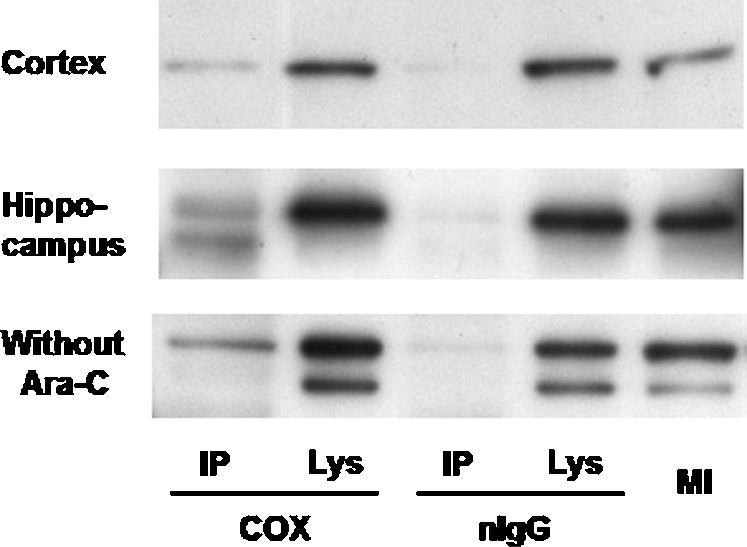
To probe for an association between mitochondrial cytochrome oxidase (COX) and lactate dehydrogenase (LDH), antibodies to COX and normal IgG (nIgG) were used to develop immunoprecipitates (IP) from Tween 20 (0.3%)-solubilized mitochondrial fractions of cortex and hippocampus primary cultures. Results were compared to those obtained on the supernatant remaining after immunoprecipitation (Sup). COX and nIgG immunoprecipitates were subsequently probed with an antibody that recognized all LDH isoforms. Mitochondrial fractions (MI) were also probed for LDH and results are presented as a positive control. LDH was coprecipitated with COX as compared to nIgG (no protein was coprecipitated with nIgG) in mitochondrial fractions derived from cortical as well as hippocampal areas of rat primary cultures. When the inhibitor to astrocyte development Ara-C was not used, COX coprecipitated LDH in mitochondrial fractions from cortical and hippocampal areas of rat primary cultures. Similar results obtained with and without Ara-C (the lack of Ara-C facilitates development of astrocytes among as neurons in cultures) indicate that astrocytes also contain mLDH.

To further explore the association between LDH and mitochondrial proteins, we probed both isolated mitochondria and COX immunoprecipitates for LDH. In contrast to normal IgG, COX significantly coprecipitated LDH in mitochondrial fractions from lysates of cells derived from cortical and hippocampal areas (Fig. 11). We used a no-AraC control to increase the relative abundance of astrocytes as occurs *in vivo*. Consequently, our results cannot be interpreted to solely reflect mLDH neuronal expression, as mLDH may be common to both neurons and astrocytes. Such a conclusion is consistent with recently reported results [24] showing that cells from a human astrocytoma cell line contain mLDH.

## Discussion

Potential importance of lactate for brain metabolism [7] and glutamatergic signaling has been proposed [10]. However, the presence of a mitochondrial lactate oxidation complex has not previously been evaluated. Now, we provide data from adult brain cross-sections and primary cell cultures supporting the presence of a mitochondrial lactate oxidation complex (mLOC) in neurons. This protein complex contains a lactate/pyruvate transport protein (MCT1 or MCT2), LDH, and COX. The neuronal lactate oxidation complex we observed would allow cerebral neurons to take up and oxidize lactate regardless of the source of lactate presentation (i.e., lactate from glycolysis in the same neuron or adjacent astrocytes, or lactate delivered by the systemic circulation). Accordingly, and consistent with the results of others [7], [10], [26], our observations would permit operation of both Cell-Cell and Intracellular Lactate Shuttles in brain. That such a functional complex exists *in vivo* is consistent with studies on human patients suffering traumatic brain injury (TBI). For instance, others [27], [28] have demonstrated that patients suffering moderate to severe TBI achieved more favorable outcomes if they were capable of net brain lactate uptake in comparison with patients demonstrating lactate release, who experienced unfavorable outcomes. Similarly, it has been demonstrated that the intravenous infusion of 100 mM L-lactate improved cognitive recovery by preserving cerebral ATP generation following TBI in rats [29].

One of the unique characteristics of neuronal lactate oxidation complex is that MCT2 is a primary monocarboxylate transporter in mitochondria, in contrast to skeletal muscle, in which MCT1 is the sole mitochondrial monocarboxylate transporter [12], [13]. The prominent expression of MCT2 in neurons is probably relevant physiologically. The lactate concentration in cerebral extracellular fluid is typically ∼1 mM [2], [27]–[31], such that it makes sense that the low-K_m_ transporter isoform (MCT2: 0.7 mM) is preferred. On the other hand, exclusive expression of MCT2 in the neuronal lactate oxidation complex could be problematic because lactate concentration in cerebral extracellular fluid can rise several-fold (∼3 mM) under physiological levels of neuronal hyperactivation [20], [27]–[31]. Hence, it makes sense that the mitochondrial lactate oxidation complex in neurons benefits from expressing both MCT1 and MCT2 isoforms. The K_m_ for MCT1 (3.5 mM) is higher than that of MCT2, so the presence of both MCT isoforms could provide metabolic flexibility across the physiological range of arterial and CSF lactate concentrations.

Equally important as the demonstration of mitochondrial MCTs was that we also detected mitochondrial LDH in rat neurons. Lactate oxidation to pyruvate is necessary to allow neuronal mitochondria to utilize lactate as an energy source [32], [33]. Although it has been controversial for skeletal muscle [34]–[36], several groups have recently provided independent evidence that LDH is located in brain mitochondria and that those mitochondria are capable of oxidizing lactate [24], [33]. For instance, Passarella and colleagues demonstrated functional lactate oxidation by means of mitochondrial LDH in rat cerebellar granule cells. Those results complement their previous observations of lactate oxidation by mitochondria from rat heart and liver [33], [37], [38]. And, most recently, using electrophoretic and immunohistochemical analyses, ^13^C-NMR, and HPLC, Lemire et al. demonstrated that mitochondria in human astrocytoma cells utilize LDH for lactate oxidation [24]. Here we showed for the first time that LDH is located in mitochondria of neurons and exists in association with COX and an MCT, both *in vivo* and *in vitro*. Along with the results of previous studies, the findings of the present study strongly suggest that the intracellular lactate shuttle (ILS) is a significant carbohydrate metabolic system in brain (especially neurons) as well as in muscles [1], [35]. In this regard we note that Schurr and Payne [39] demonstrated that malonate and oxamate, two different LDH inhibitors, significantly suppressed lactate-supported neuronal function, but did not inhibit pyruvate-supported neuronal function in rat hippocampal slices. On that basis they proposed a neuronal ILS [39].

The presence of a neuronal ILS [39] does not obviate the presence of an ANLS because astrocytes also contain mitochondrial LDH and effectively oxidize lactate [24]. The contribution of astrocytes to neuronal mitochondrial lactate consumption is estimated to be approximately 15% [39]. However, we can conclude that a neuronal ILS is a crucial metabolic system for brain to carry out ANLS metabolism. Additionally, this ILS, based on a neuronal lactate oxidation complex, would emphasize the role of mitochondrial redox in creating the proton and lactate anion concentration gradients necessary for the oxidative disposal of lactate in the mitochondrial reticulum in neurons, subsequently providing neuroprotective effects against previously described excitotoxicity [39], [40].

Interestingly, distribution of LDH-M and LDH-H varied across the brain. LDH-M is expressed prominently in the cortex, while LDH-H is expressed strongly in the hippocampus. Because our results differ somewhat from those expected on the basis of previously reported studies assessing LDH mRNA expression by *in situ* hybridization, where LDH-H mRNA was predominant in cortex [41], it may be that regional LDH expression in the brain is regulated post-transcriptionally.

The results of the present study cannot provide a physiological explanation for the observed differences in regional distribution of LDH isoforms. In skeletal muscle, white, fast-twitch glycolytic fibers express mainly LDH-M and act as lactate producers, while red, slow oxidative muscle fibers express significantly more LDH-H and act as lactate consumers [6]. Hence, along with differences in mitochondrial content, fast white and slow red fibers establish the lactate concentration gradient allowing the Cell-Cell Lactate Shuttle to operate in muscle tissue. However, in the brain, cells in both cortex and hippocampus contain elaborate mitochondrial networks and are metabolically active [42]. The presence of mLDH is necessary to accomplish the first step in lactate oxidation, but the specific isoform present appears to be of secondary importance and tracks that expressed in the specific tissue or cell type under consideration. For example, the heart is typically a net lactate consumer and contains the LDH-H isoform. However, the liver—typically also a net lactate consumer—contains mostly the isoform prominent in white skeletal muscle, LDH-M. As well, mitochondria of astrocytes contain LDH-H and effectively oxidize lactate [24], but the ANLS posits that astrocytes release lactate for oxidation in neurons. Consequently, for the present, we consider the existence of both LDH-M and LDH-H isoforms in neuronal mitochondria to permit both sensitivity and metabolic flexibility of shuttling in response to changing lactate levels. By these means, astrocytes may oxidize lactate generated from glycolysis or they may release lactate for consumption by neurons. As well, irrespective of an ANLS, both astrocytes and neurons may utilize lactate provided form the systemic circulation when arterial [lactate] rises as occurs under exercise [9] and other stresses [27].

In summary, we provide evidence supporting the concept of lactate shuttling within neurons and between neurons and glia. Others have established that lactate uptake and oxidation are characteristics of intact brains, brain slices, and isolated cells and mitochondria. In the present report, we show how cerebral and neuronal lactate uptake and oxidation might occur. As it does in skeletal muscle, it is possible that the mitochondrial reticulum in neurons establishes the concentration gradients allowing intracerebral use of lactate as an energy source. The presence of an mLOC in neurons would similarly establish the concentration gradients allowing for cell-cell lactate exchange and permitting use of vascularly delivered lactate. Along with our own previous results as well as those of others showing lactate oxidation *in vivo*, it appears that the present results support generality of the utility of lactate shuttling in energy-substrate exchange and metabolic signaling [35]. These findings may encourage development of new approaches to support nutrient delivery to a stunned brain following trauma.
